# Education and training of medical physicists in South East Asia: accomplishments and challenges†

**DOI:** 10.2349/biij.7.3.e23

**Published:** 2011-07-01

**Authors:** BJ Thomas

**Affiliations:** Faculty of Science and Technology, Queensland University of Technology, Brisbane Queensland, Australia

**Keywords:** John Cameron, Education, Clinical Training, Medical Physics, South East Asia

## Abstract

John Cameron has made significant contributions to the field of Medical Physics. His contributions encompassed research and development, technical developments and education. He had a particular interest in the education of medical physicists in developing countries. Structured clinical training is also an essential component of the professional development of a medical physicist. This paper considers aspects of the clinical training and education of medical physicists in South-East Asia and the challenges facing the profession in the region if it is to keep pace with the rapid increase in the amount and technical complexity of medical physics infrastructure in the region.

## INTRODUCTION

John Cameron commenced his career at a time when there were relatively few medical physicists in the USA. He headed the medical physics programme at the University of Wisconsin for three decades. This was the first medical physics programme in the USA to gain departmental status. Many well-known medical physicists benefited greatly from his mentoring. He made many significant contributions to the profession of medical physics, particularly in the areas of research and development (R&D) and education.

Perhaps his most notable R&D achievements were his contributions to thermoluminescence dosimetry, the invention of bone densitometry and the development of the Wisconsin test cassette. Apart from his contributions to the profession in the USA he also found time to promote the profession outside the USA. He has been credited with many insightful quotations. Perhaps his most well-known quotation is one related to R&D, “Anything worth doing is worth doing poorly,” meaning, “It doesn't have to be perfect, just get it to work!”. To which should be added “perfection can come later”.

His contributions were recognised by others in the field and he was the recipient of many prestigious awards including the Coolidge Award from the AAPM and the Madam Curie Award from the IOMP.

The John Cameron Memorial Lecture was inaugurated at the 3^rd^ SEACOMP conference held in Malaysia (2000). On the occasion of the 5^th^ Memorial Lecture, I have chosen the topic of “Education and Training of Medical Physicists in SE Asia-Accomplishments and Challenges”.

## THE CURRENT SITUATION

South-East Asia, like many other regions in the world, has experienced a rapid increase in the amount and complexity of equipment used in therapy and diagnosis. There is also an expectation of improved healthcare in the region. These factors have resulted in an increased demand for medical physicists in the region. However, the education and clinical training programmes have not kept pace with this demand. The number of medical physicists in six of the region’s nations (combined population of approximately 550 million) was about 250 in 2006/2007 [1] compared with some 5000 in the USA (population of approximately 300 million). Asia is estimated to have a shortage of about 6000 medical physicists [2]. However, the shortage of medical physicists is not unique to Asia. It is a worldwide problem. The response by the nations of SE Asia to address this shortage has been somewhat varied.

SE Asia has a number of high-quality Masters programmes in medical physics. (see ref. [3] for a list of MSc programmes in medical physics in SE Asian countries). Graduates from these programmes are well-prepared (academically) to enter clinical training programmes. However some countries do not have medical physics Masters programmes and degree graduates, with limited education in medical physics, are often appointed to junior medical physics positions. They are expected to acquire their knowledge of medical physics “on the job”, often in a relatively unstructured manner. As stated above, even in those countries which do have Masters programmes in medical physics, the numbers graduating from the Masters programmes have not always kept up with the demand for medical physicists.

Perhaps the greater problem has been the lack of structured clinical training programmes in the nations. Until 2006, most nations relied upon learning “on the job” for medical physicists to acquire clinical competence. In some cases the clinical training was directed towards technical competence rather than professional competence. Even now, in 2010, not all nations have adopted structured clinical training programmes and those that have are yet to establish a **nationally**-accepted standard of clinical training.

## CHALLENGES OF THE NEXT DECADE

The challenges of the next decade reflect the need to significantly increase the number of clinically-qualified medical physicists in the region. By clinically-qualified, I mean that they meet the requirements of:

• a basic degree in physics (or equivalent);
• a postgraduate degree in medical physics; and
• at least two years of supervised, structured clinical training.

To meet these requirements the nations need to establish more postgraduate programmes in medical physics. They also need to establish sustainable, structured programmes of clinical training.

Important to meeting these needs is the necessity to attract high-quality students initially into undergraduate degree programmes in physics. This can be difficult because of the shortage of physics students and teachers, a problem which has been recognised for some time but not adequately addressed in some nations [4, 8]. It is important that careers in physics (in general) and medical physics (in particular) are promoted as attractive options to university and secondary school students. The professional body, representing medical physicists, has an important role to play in promoting such careers.

The RCA/IAEA has developed guides for clinical training programmes in the specialties of radiation oncology medical physics (ROMP) [5], diagnostic radiology medical physics (DRMP) [6], and nuclear medicine physics (NMP) [7]. These have been adopted by some nations in the region. Table 1 lists the pilots of the RCA/IAEA clinical training programmes which are underway or have been completed. At least two other nations have indicated a desire to proceed with pilot clinical training programmes.

**Table 1:** Pilot RCA/IAEA clinical training programs in medical physics (in progress or completed).

**Clinical Training Programs in**	**Nation**	**Commenced**
ROMP	Thailand The Philippines Malaysia	2007 (completed) 2009 2011
DRMP	The Philippines Thailand	2010 2010
NMP	Bangladesh Thailand	2010 2011

It is worth noting that any nation may use all or part of the documentation of the RCA/IAEA clinical training programmes without formally participating in a pilot trial. It is anticipated that nations will continue with a structured programme of clinical training once the initial trial is complete and ideally establish a national and perhaps regional standard of clinical training for medical physicists in the three discipline areas. This brings me to another challenge for the nations in the next decade – overcoming the problems associated with distance.

On many occasions I have heard the statement that “this department is too far from the major centre to be included in a training programme”. My response to that statement is that I believe that medical physicists in remote areas have a need to be as clinically competent as their colleagues in the major centres. Often the medical physicists in remote centres are sole practitioners in the department without immediate support from colleagues. In order to contribute to a high quality of healthcare, they must have the opportunity to develop their knowledge and skills. How can medical physicists practise independently if their education and training are not thorough and comprehensive? As John Cameron stated, “The growth of technology is such that it is not possible today for a nuclear physicist to switch into medical physics without training. The field is now much more technical. More training is needed to do the job.”

Retention of clinically-qualified medical physicists is also important if adequate numbers are to be available to service the needs of the healthcare system in a country. Adequate remuneration is an important factor in retaining staff, particularly when salaries offered overseas may be significantly higher. However, there are other factors which impact upon retention, such as a lack of status and recognition by clinical colleagues, workload and lack of support for continuing professional development [9].

A major challenge of the next decade is to ensure the viability of the medical physics professional bodies in the region. Not all nations have well-established professional bodies and yet these bodies are essential to setting the standards expected of medical physicists. They also play a role in ensuring that these standards are acquired and potentially in certifying those medical physicists who pass an exam to demonstrate their competence.

In summary, I believe that the region is at the start of an exciting decade of development in the area of medical physics. There are many challenges but also many associated opportunities. I anticipate that whoever presents the John Cameron Memorial Lecture in, say 2015, will be able to report on significant advances in the education and training of medical physicists in the region.
